# Cognitive profiles in childhood and adolescence differ between adult psychotic and affective symptoms: a prospective birth cohort study

**DOI:** 10.1017/S0033291717000393

**Published:** 2017-10-09

**Authors:** S. Koike, J. Barnett, P. B. Jones, M. Richards

**Affiliations:** 1 MRC Unit for Lifelong Health and Ageing at UCL, 33 Bedford Place, London WC1B 5JU, UK; 2 University of Tokyo Institute for Diversity & Adaptation of Human Mind (UTIDAHM), 3-8-1 Komaba, Meguro-ku, Tokyo 153-8902, Japan; 3 Center for Evolutionary Cognitive Sciences, Graduate School of Arts and Sciences, The University of Tokyo, 3-8-1 Komaba, Meguro-ku, Tokyo 153-8902, Japan; 4 Department of Psychiatry, University of Cambridge, Cambridge CB2 0SZ, UK; 5 Cambridge Cognition Ltd, Cambridge CB25 9TU, UK; 6 CAMEO, Cambridgeshire & Peterborough NHS Foundation Trust, Cambridge CB21 5EF, UK

**Keywords:** Anxiety, birth cohort studies, cognitive development, depression, schizophrenia

## Abstract

**Background:**

Differences between verbal and non-verbal cognitive development from childhood to adulthood may differentiate between those with and without psychotic symptoms and affective symptoms in later life. However, there has been no study exploring this in a population-based cohort.

**Method:**

The sample was drawn from the MRC National Survey of Health and Development, and consisted of 2384 study members with self-reported psychotic experiences and affective symptoms at the age of 53 years, and with complete cognitive data at the ages of 8 and 15 years. The association between verbal and non-verbal cognition at age 8 years and relative developmental lag from age 8 to 15 years, and both adult outcomes were tested with the covariates adjusted, and mutually adjusted for verbal and non-verbal cognition.

**Results:**

Those with psychotic experiences [thought interference (*n* = 433), strange experience (*n* = 296), hallucination (*n* = 88)] had lower cognition at both the ages of 8 and 15 years in both verbal and non-verbal domains. After mutual adjustment, lower verbal cognition at age 8 years and greater verbal developmental lag were associated with higher likelihood of psychotic experiences within individuals, whereas there was no association between non-verbal cognition and any psychotic experience. In contrast, those with case-level affective symptoms (*n* = 453) had lower non-verbal cognition at age 15 years, and greater developmental lag in the non-verbal domain. After adjustment, lower non-verbal cognition at age 8 years and greater non-verbal developmental lag were associated with higher risk of case-level affective symptoms within individuals.

**Conclusions:**

These results suggest that cognitive profiles in childhood and adolescence differentiate psychiatric disease spectra.

## Introduction

Epidemiological studies show that lower cognition in childhood is associated with the manifestation of schizophrenia (Jones *et al.* 1994; Cannon *et al.* 2002*b*; Zammit *et al.* 2004; Koenen *et al.* 2009; Welham *et al.* 2009; Reichenberg *et al.* 2010; Khandaker *et al.* 2011). This is in line with clinical case–control studies for first-episode psychosis as well as clinical high risk for psychosis (Fusar-Poli *et al.* 2012; Giuliano *et al.* 2012). Lower childhood cognitive function is also observed in people with self-reported psychotic experiences (Horwood *et al.* 2008; Barnett *et al.* 2012; Gur *et al.* 2014; Khandaker *et al.* 2014, Nishida *et al.* 2014), suggesting that investigating the broader psychosis spectrum may help to understand the pathophysiology of schizophrenia. However, cognitive impairment in childhood also occurs with other psychiatric disorders such as major depression and self-report affective symptoms, suggesting common risk pathways (van Os *et al.* 1997; Zammit *et al.* 2004; Hatch *et al.* 2007; Koenen *et al.* 2009; Meier *et al.* 2014; Rock *et al.* 2014; Trivedi & Greer, 2014).

**Fig. 1.**
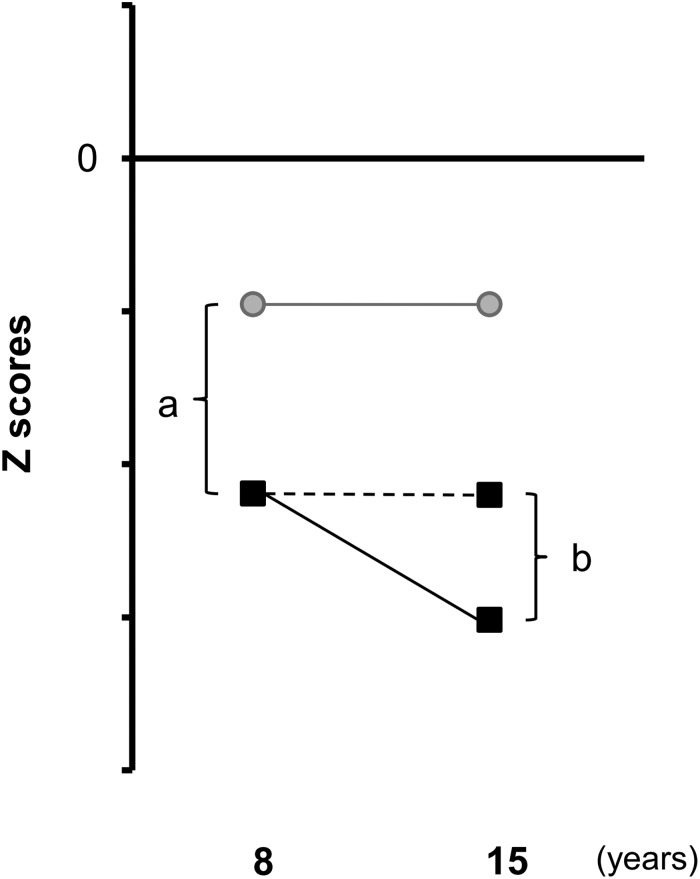
Example of cognitive development in the two domains. Cognitive development using *Z*-scores of the two domains in this study is illustrated. Gap a indicates cognitive discrepancy between the two domains at age 8 years. Gap b indicates relative cognitive lag during adolescence, hypothesizing no difference in *Z*-scores through development within an individual (dashed line). Black line and square dots represent verbal cognition, and grey line and circle dots represent non-verbal cognition.

Some longitudinal epidemiological studies have suggested that a developmental deficit in childhood (Reichenberg *et al.* 2010) or a developmental lag (delay of typical development) through adolescence (Fuller *et al.* 2002; MacCabe *et al.* 2013) in verbal cognition differentiates those with schizophrenia from healthy participants, whereas verbal developmental lag does not appear to differentiate affective disorders (Cannon *et al.* 2006; Reichenberg *et al.* 2010). The former is also observed in children with dyslexia who are likely to have psychotic experiences in adolescence (Khandaker *et al.* 2014). Although still controversial (Carrion *et al.* 2011; Lin *et al.* 2013), clinical high-risk studies for psychosis showed that lower scores in verbal memory and verbal fluency tests could predict the later onset of psychosis (Giuliano *et al.* 2012), suggesting that developmental deficits and/or lag in verbal cognition play a key role in the emergence of psychotic symptoms. In contrast, epidemiological studies showed that people with affective symptoms have no specific developmental deficit and lag pattern in childhood (Reichenberg *et al.* 2010) and adulthood (Meier *et al.* 2014) in verbal and non-verbal cognition. Examining deficits and adolescent developmental lag in verbal and non-verbal cognition, and within-individual cognitive discrepancy between the two cognitive domains between those with and without psychological symptoms in later life would test the specificity of the neurocognitive (and thus neurodevelopmental) model of psychosis and depression spectra (Fig. 1) (van Os, 2013). However, to the best of our knowledge, there has been no prospective cohort study that has examined whether relative verbal cognitive developmental lag in the general population is associated with adult psychotic experiences, and, conversely, whether non-verbal lag is associated with adult affective symptoms.

The Medical Research Council (MRC) National Survey of Health and Development (NSHD) is one of the longest continuously followed prospective birth cohort studies in the world, beginning in 1946 in England, Scotland and Wales (Wadsworth *et al.* 2006). Studies based on this cohort have reported cognitive delay in clinical cases of schizophrenia (Jones *et al.* 1994) and depression (van Os *et al.* 1997), as well as broader spectra of psychotic experiences (Barnett *et al.* 2012; Nishida *et al.* 2014) and affective symptoms (Hatch *et al.* 2007). Although symptoms in self-report questionnaires might have a different distribution to those from clinical interviews, the former are likely to be more representative at the general population level with broader disease spectra. In the present study, we extended this by examining psychosis and depression spectra in relation to deficits and adolescent developmental lag in verbal and non-verbal cognition. We hypothesized that a specific developmental deficit and lag in verbal cognition is associated with psychotic experiences in adulthood, but not with affective symptoms.

## Method

### Participants

The NSHD is one of the longest large prospective cohort studies, originally consisting of 5362 randomly selected children from all single births within marriage during 1 week in March 1946 in England, Scotland and Wales. Of the original sample, 3820 participants had complete cognitive data at ages 8 and 15 years. When study members were aged 53 years, they reported psychotic experiences and depressive symptoms in self-reported questionnaires. Of 3035 participants who were interviewed at that age, 2923 and 2920 participants provided information on psychotic experiences and affective symptoms (see below). Reasons for non-response at age 53 years were 469 deaths, 640 permanent refusals, 580 non-residents and 638 failures to contact. However, the responding participants were broadly representative of the national population of similar age (Wadsworth *et al.* 2003). Finally, one or more adult psychiatric outcomes were available for 2384 study members with complete cognitive data. Those with complete data were more likely to be female, had heavier birth weight, and higher cognition at ages 8 and 15 years than those with missing data (*p* < 0.05).

Ethical approval for this study was obtained from the North Thames Multicentre Research Ethics Committee. All participants gave written informed consent.

### Measures

#### Psychological symptoms in adulthood

Psychotic experiences were self-reported over the previous 12 months at age 53 years using the five-item version of the Psychosis Screening Questionnaire (PSQ) (Bebbington & Nayani, 1995; Johns *et al.* 2004) including three delusional (thought interference, persecution, and strange experiences), one hallucinatory and one hypomanic questions. All items were answered as either ‘no’, ‘unsure’ or ‘yes’. In the original PSQ each of these questions has at least one follow-up question to elicit further detail; however, due to time constraints these were not asked in the NSHD. We therefore defined ‘yes’ for each leading question as the existence of a symptom and the other responses as negative. As in a previous study using this cohort (Nishida *et al.* 2014), more than half of the respondents gave a positive response to the hypomania item ‘Have there been times when you felt very happy without a break for days on end?’. Further, 643 respondents (22.0%) rated themselves as ‘unsure’ on this item. In addition, there has been a biological and cognitive debate over whether mania and hypomania should be included within the psychosis or affective spectrum (Trotta *et al.* 2015; Bora, 2016). Therefore, consistent with the previous study, we did not use this item in the analysis.

Self-reported affective symptoms were measured at age 53 years using the 28-item version of the General Health Questionnaire (GHQ-28) (Goldberg & Hillier, 1979). Each item was scored using a four-level Likert scale for total symptom score, and was recoded into 0-0-1-1 for deriving a diagnostic risk threshold (‘caseness’). This threshold was defined as a score of 5 or more on this summed recoded score (range 0–28), which was validated using the Clinical Interview Schedule (Goldberg & Hillier, 1979). The GHQ-28 consists of four components (somatic symptoms, anxiety and insomnia, social dysfunction, and severe depression) (Goldberg & Hillier, 1979). To test possible differential effects of these types of affective symptoms, we set thresholds for each subscale at the top 10% score, which matched the prevalence of GHQ-28 caseness and that of least one subscale.

#### Cognition in childhood and adolescence

Cognition at age 8 years was measured at school using three verbal tests (reading comprehension, word reading, and vocabulary) and one non-verbal test (picture intelligence, which consisted of three tasks: odd-one out, sequence detection and abstract reasoning) devised by the National Foundation for Educational Research (Pigeon, 1964). Cognition at age 15 years was measured at school using three verbal tests (Watts Vernon sentence completion, mathematics, and the verbal section of the Alice Heim Group Ability Test), and one non-verbal test (reasoning) using the non-verbal section of the Alice Heim Group Ability Test (Heim, 1970). The non-verbal tests at both ages had some similarity with the matrix reasoning and picture concepts subtests of the Wechsler Intelligence Scale for Children (Kaplan & Saccuzzo, 2013). Non-verbal cognition also involves processing speed and is thought to be less dependent on educational and cultural learning than verbal cognition (Kaplan & Saccuzzo, 2013). As verbal and non-verbal cognition was measured by different tests at ages 8 and 15 years, we calculated standardized verbal and non-verbal scores at both ages by summing each standardized score. We also calculated a relative developmental lag score for verbal and non-verbal cognition as the relevant *Z*-score at age 15 years minus corresponding *Z*-score at 8 years; thus a negative value represents relative decrease in scores from age 8 to 15 years, i.e. increasing developmental lag with respect to peers (Fig. 1). The ages 8 and 15 years were, respectively, the earliest and latest that cognition was tested during development. Thus this interval maximized the potential for capturing any effect of cognitive lag.

#### Potential confounding variables

Based on previous studies with this cohort the following potential confounders were selected: sex, birth weight, birth order, mother's education, and social class of origin (at age 11 years or, if this was unknown, at age 4 or 15 years).

Mother's education was dichotomized into primary only or above, and family social class was classified into three categories (professional or intermediate, skilled non-manual, and skilled manual or unskilled).

### Statistical analysis

We initially used χ^2^ tests and *t* tests, as appropriate, to test for differences between the independent variables in those with and without a positive response on the adult mental health outcomes [each individual PSQ item (all coded as positive or negative) or GHQ-28 ‘caseness’]. Multivariable logistic regression was then used to test associations between childhood and adolescent cognition and the outcomes, using verbal and non-verbal cognition at age 8 years and verbal and non-verbal developmental lag from age 8 to 15 years as independent variables. Outcomes were coded as separate for each PSQ item and for GHQ-28 caseness. We also tested whether cognitive characteristics were associated with the number of positive PSQ responses using multivariate regression analysis. As several participants who reported the existence of psychotic experiences also had affective symptoms (53.9%), we also grouped into psychotic experience only (PE only), affective symptoms only (AFF only), and both, with each of these groups compared with those with neither psychotic experience nor affective symptoms (no symptom) as the reference category. In addition, we compared the PE-only group with the AFF-only group to directly investigate differences in cognitive profiles. In this second analysis, psychotic experience presence was defined as one or more psychotic experience items of strange experience and hallucination, because previous studies showed a relatively low degree of positive responses that could represent more definitive characteristics of a psychosis spectrum (Johns *et al.* 2004; Nishida *et al.* 2014). For all multivariable models, we mutually adjusted verbal and non-verbal cognition. In addition, to test for possible synergy between verbal and non-verbal cognitive domains increasing the severity of outcomes, we tested interactions between verbal and non-verbal cognition at age 8 years and lag from age 8 to 15 years. Since verbal and non-verbal cognition are positively correlated (age 8 years: *r* = 0.57, *p* < 0.001; age 15 years: *r* = 0.61, *p* < 0.001), we tested multicollinearity using variance inflation factors (VIF) for mutual adjustment of cognitive variables. No collinearity was defined by less than 10 of each VIF value for the independent variable.

Statistical significance was set at 0.05, and all analyses were conducted using SPSS Statistics 22 (IBM Corp., USA).

## Results

Overall, the no-symptom group had stable *Z*-transformed cognitive scores from age 8 to 15 years in verbal and non-verbal domains (Table 1).

**Table 1.** Cognitive characteristics in childhood and adolescence on each psychotic experience and affective symptoms

	PSQ										GHQ-28	
	Thought interference		Persecution		Strange experience		Hallucination		Any of psychotic experiences		Affective symptoms^a^	
	No or unsure	Yes	No or unsure	Yes	No or unsure	Yes	No or unsure	Yes	No or unsure	Yes	Non-case	Caseness
Participants, *n* (%)	1945 (81.8)	433 (18.2)	1870 (78.6)	508 (21.4)	2083 (87.6)	296 (12.4)	2289 (96.3)	88 (3.7)	1607 (67.5)	772 (32.5)	1913 (80.9)	453 (19.1)
Male, *n* (%)	959 (82.7)	200 (17.3)	905 (78.2)	253 (21.8)	1001 (86.4)	158 (13.6)	1105 (95.4)	53 (4.6)*	772 (66.6)	387 (33.4)	998 (86.6)	154 (13.4)***
Cognition at age 8 years												
Verbal domain	0.10 (0.99)	−0.14 (0.92)***	0.07 (0.99)	0.00 (0.97)	0.07 (0.98)	−0.09 (0.99)**	0.07 (0.98)	−0.29 (0.96)***	0.11 (0.99)	−0.06 (0.95)***	0.06 (0.98)	0.05 (0.95)
Non-verbal domain	0.11 (0.94)	−0.07 (1.01)***	0.08 (0.95)	0.03 (0.98)	0.09 (0.94)	−0.07 (1.05)*	0.08 (0.95)	−0.24 (1.08)**	0.11 (0.93)	−0.00 (1.00)**	0.09 (0.95)	0.04 (0.93)
Cognition at age 15 years												
Verbal domain	0.12 (0.96)	−0.05 (0.89)***	0.10 (0.95)	0.05 (0.95)	0.12 (0.94)	−0.14 (0.98)***	0.10 (0.94)	−0.32 (1.00)***	0.13 (0.95)	0.00 (0.94)**	0.10 (0.94)	0.06 (0.95)
Non-verbal domain	0.09 (0.94)	−0.06 (0.95)**	0.07 (0.93)	0.03 (1.00)	0.08 (0.93)	−0.06 (1.05)*	0.07 (0.94)	−0.14 (1.15)	0.09 (0.93)	−0.00 (0.98)*	0.10 (0.93)	−0.10 (0.97)***
Developmental lag from age 8 to 15 years^b^												
Verbal domain	0.024 (0.702)	0.093 (0.682)	0.033 (0.703)	0.051 (0.683)	0.048 (0.703)	−0.048 (0.662)*	0.038 (0.702)	−0.026 (0.611)	0.023 (0.702)	0.064 (0.690)	0.045 (0.706)	0.009 (0.667)
Non-verbal domain	−0.019 (1.015)	0.016 (1.118)	−0.016 (1.027)	0.000 (1.062)	−0.016 (1.030)	0.008 (1.067)	−0.017 (1.030)	0.098 (1.136)	−0.020 (1.005)	0.002 (1.093)	0.019 (1.042)	−0.142 (0.980)**

Data are given as mean (standard deviation) unless otherwise indicated.

PSQ, Psychosis Screening Questionnaire; GHQ-28, 28-item version of the General Health Questionnaire.

Value significantly different from the no or unsure participants: * *p* < 0.05, ** *p* < 0.01, *** *p* < 0.001 (by χ^2^ test for sex, and *t* test for the other variables).

a Differences in existence of GHQ-28 subscale cases are shown in online Supplementary Table S1.

b Differences in *Z*-scores from age 8 to 15 years for verbal and non-verbal domains (score at age 15 years – 8 years). A positive score represents an increase of developmental lag from the mean.

### Associations with adult psychotic experiences

The rates of positive respondents for each psychotic experience items varied between 3.7% and 21.4%, ‘hallucination’ was the lowest and ‘persecution’ the highest (Table 1). In comparing those with and without each psychotic experience, those with lower cognition in at least one domain were more likely to have psychotic experiences for all PSQ items at age 53 years except ‘persecution’. These associations remained significant after adjusting for confounding variables (online Supplementary Table S2).

**Fig. 2.**
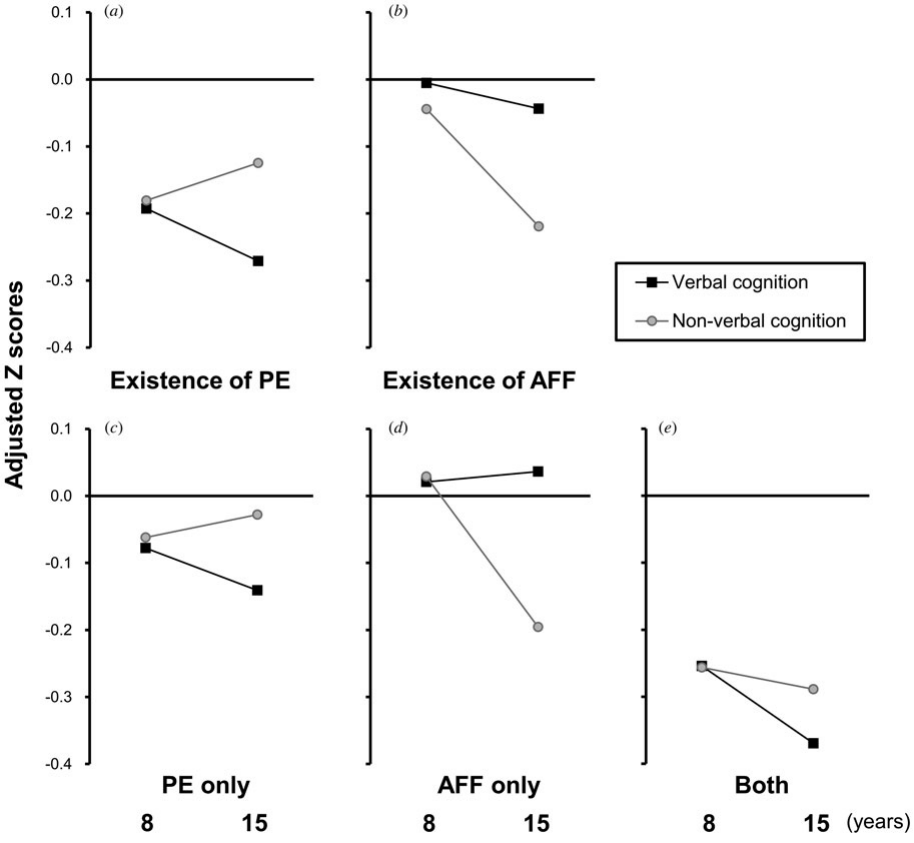
Cognitive development patterns by existence of psychotic experiences (PE) and affective symptoms (AFF). (*a, b*) Characteristics of cognitive scores in those with PE (and AFF) are illustrated using adjusted *Z*-transformed scores for sex and mother's education, and for those without PE (and AFF) as zero. (*c*–*e*) Characteristics of three different subgroups (PE only, AFF only, and both symptoms) are also illustrated using adjusted *Z*-transformed scores, with the no-symptom group as zero.

After mutual adjustment for verbal and non-verbal cognitive variables (maximum VIF = 2.2), lower verbal cognition at age 8 years was associated with higher likelihood of each PSQ symptom except for ‘persecution’, whereas no significant association between non-verbal cognition and any of these psychotic experiences was evident (Table 2). In addition, greater verbal developmental lag from age 8 to 15 years was associated with higher likelihood of the psychotic experiences ‘strange experience’ and ‘hallucination’, whereas, again, there was no association between non-verbal lag and any of these psychotic experiences. In sum, those with relatively lower verbal cognition in childhood and adolescent verbal developmental lag, which resulted in wider cognitive discrepancy with relatively lower verbal cognition during adolescence, were more likely to have psychotic experiences (Fig. 2*a*).

**Table 2.** Association of cognitive characteristics in childhood and adolescence with each psychotic experience and affective symptoms^a^

	PSQ					GHQ-28
Independent variables	Thought interference (*n* = 433 *v*. 1945)	Persecution (*n* = 508 *v*. 1870)	Strange experience (*n* = 296 *v*. 2083)	Hallucination (*n* = 88 *v*. 2289)	Any of psychotic experiences (*n* = 772 *v*. 1607)	Affective symptoms (*n* = 453 *v*. 1913)
Cognition at age 8 years						
Verbal domain	0.81 (0.68–0.97)*	0.92 (0.78–1.09)	0.79 (0.64–0.97)*	0.55 (0.38–0.80)**	0.82 (0.70–0.94)**	1.05 (0.88–1.26)
Non-verbal domain	0.91 (0.76–1.10)	1.00 (0.84–1.19)	0.99 (0.80–1.22)	1.02 (0.70–1.47)	0.99 (0.85–1.15)	0.77 (0.64–0.93)**
Developmental lag from age 8 to 15 years						
Verbal domain	1.07 (0.88–1.31)	0.99 (0.83–1.20)	0.71 (0.56–0.90)**	0.52 (0.34–0.79)**	0.98 (0.83–1.15)	1.11 (0.91–1.36)
Non-verbal domain	0.93 (0.80–1.08)	1.00 (0.87–1.14)	1.04 (0.88–1.24)	1.23 (0.91–1.67)	0.98 (0.87–1.10)	0.74 (0.64–0.86)***

Data are given as odds ratio (95% confidence interval).

PSQ, Psychosis Screening Questionnaire; GHQ-28, 28-item version of the General Health Questionnaire.

a This model was adjusted by the confounding variables of sex, birth weight, birth order, mother's education, and childhood social class, and the independent variables of cognition at age 8 years and developmental lag from age 8 to 15 years in verbal and non-verbal domains. Unadjusted and other adjusted models, and models for GHQ-28 subscale cases are shown in online Supplementary Table S2.

Significant coefficient: * *p* < 0.05, ** *p* < 0.01, *** *p* < 0.001.

### Associations with adult affective symptoms

For case-level affective symptoms, 19.1% (453/2376) participants were identified as cases. Frequencies of case-level somatic symptoms, anxiety and insomnia, social dysfunction and severe depression were 180 (7.6%), 214 (9.0%), 219 (9.2%) and 182 (7.7%), respectively (online Supplementary Table S1). There were 450 (19.0%) study members with case-level symptoms for at least one subscale. Those with case-level symptoms had lower non-verbal cognition at age 15 years, and greater developmental lag in the non-verbal domain (Table 1). For verbal cognition, however, there was no association between those with and without affective symptoms.

After mutually adjusting for cognitive variables, lower non-verbal cognition at age 8 years and greater non-verbal developmental lag were associated with higher risk of GHQ-28 caseness (Table 2). There was no association between verbal cognitive scores and GHQ-28 caseness. These trends were similar for GHQ-28 subscore cases (online Supplementary Table S2). Multivariate regression analysis also showed that lower verbal cognition at age 8 years was associated with the number of positive responses in the PSQ at age 53 years (B = −0.090, s.e. = 0.033, *p* = 0.007). In sum, those with relatively lower non-verbal cognition in childhood and adolescent non-verbal developmental lag, which resulted in wider cognitive discrepancy with relatively lower non-verbal cognitions, were more likely to have affective symptoms (Fig. 2*b*).

### Associations with adult psychotic experiences and affective symptoms

Sample sizes for the grouped outcomes were 194 for PE only (8.2%), 315 for AFF only (13.3%), 138 for both (5.8%) and 1714 for the no-symptom group (72.6%). In logistic regression models with the no-symptom group as the reference, the AFF-only group showed the characteristics of the cognitive discrepancy; a smaller verbal developmental lag (i.e. relatively better development in verbal cognition), lower non-verbal cognition at age 8 years, and a greater non-verbal developmental lag (Table 3). These trends are illustrated in Fig. 2*c*–*e*, particularly the non-verbal developmental lag in the AFF-only group and greater cognitive discrepancy (non-verbal < verbal), while the PE-only group had a developmental lag in verbal cognition and greater cognitive discrepancy (verbal < non-verbal).

**Table 3.** Cognitive characteristics in childhood and adolescence by existence of psychotic experience and/or affective symptoms^a^

Independent variables	PE only (*n* = 194) *v.* no symptoms (*n* = 1714)	AFF only (*n* = 315) *v.* no symptoms (*n* = 1714)	Both (*n* = 138) *v.* no symptoms (*n* = 1714)	AFF only (*n* = 315) *v*. PE only (*n* = 194)
Cognition at age 8 years				
Verbal domain	0.79 (0.61–1.02)	1.21 (0.98–1.49)	0.72 (0.53–0.97)*	0.64 (0.45–0.91)*
Non-verbal domain	1.12 (0.86–1.46)	0.73 (0.58–0.91)**	0.85 (0.63–1.15)	1.40 (0.98–2.01)
Developmental lag from age 8 to 15 years				
Verbal domain	0.75 (0.57–1.00)	1.33 (1.05–1.68)*	0.70 (0.50–0.99)*	0.59 (0.40–0.86)**
Non-verbal domain	1.12 (0.91–1.38)	0.66 (0.55–0.79)***	0.93 (0.72–1.18)	1.50 (1.13–2.01)**

Data are given as odds ratio (95% confidence interval).

PE, Psychotic experience; AFF, affective symptoms.

a Participants were grouped into PE-only (defined by the existence of one or more items of strange experience and hallucination), AFF-only, both, and no-symptom groups. This model was adjusted by the confounding variables of sex, birth weight, birth order, mother's education and childhood social class, and the independent variables of cognition at age 8 years and developmental lag from age 8 to 15 years in verbal and non-verbal domains. Unadjusted and other adjusted models are shown in online Supplementary Table S3.

Significant coefficient: * *p* < 0.05, ** *p* < 0.01, *** *p* < 0.001.

### Interactions between verbal and non-verbal cognition on adult psychological symptoms

When we added interactions between verbal and non-verbal cognitive domains at age 8 years and developmental lags from age 8 to 15 years to the mutually adjusted models, we found a significant interaction between verbal and non-verbal cognition at age 8 years on ‘thought interference’ at age 53 years [odds ratio (OR) = 0.87, 95% confidence interval (CI) 0.78–0.99, *p* = 0.029), with the association between lower verbal cognition at age 8 years and PE similar to the above results. Thus, greater cognitive discrepancy (verbal < non-verbal) in addition to lower verbal cognition at age 8 years was additively associated with adult ‘thought interference’.

Compared with the ‘no’-symptom group, the interaction between verbal and non-verbal cognitive lags from age 8 to 15 years was also significant in the ‘both’-symptom group (OR = 0.68, 95% CI 0.52–0.90, *p* = 0.007), with a similar effect to the above results of lower verbal cognition at age 8 years and greater verbal cognitive lag. Thus, greater discrepancy between verbal and non-verbal cognitive lags from age 8 to 15 years (verbal < non-verbal) was associated with the simultaneous existence of psychotic and affective symptoms at age 53 years.

## Discussion

A population-representative prospective birth cohort study revealed a developmental verbal–non-verbal differentiation in regard to the presence of adult psychotic experiences and affective symptoms. Low verbal cognition at age 8 years and relative developmental lag in this domain between age 8 and 15 years were associated with psychotic experiences at age 53 years, independently of non-verbal cognition and confounding factors. In contrast, lower non-verbal cognition at age 8 years and relative developmental lag in this between age 8 and 15 years were associated with risk of common mental disorder in adulthood, as assessed by the GHQ-28 caseness threshold, independently of verbal cognition and the other covariates. Participants with case-level affective symptoms but no psychotic experience additionally had higher verbal cognition at age 8 years, and rather better verbal development between the ages of 8 and 15 years. To the best of our knowledge, this is the first investigation of its kind in a population-representative prospective birth cohort study.

Strengths of this study include a large population-based prospective birth cohort, the availability of cognition objectively assessed in childhood and adolescence, and the availability of a wide range of potential confounders. In addition, since psychotic experiences are less common in midlife than in adolescence or early adulthood (van Os *et al.* 2009), these symptoms at age 53 years may be associated with more distinct cognitive profiles than those reported at a younger age (Nishida *et al.* 2014). The childhood and adolescent cognitive characteristics in those with adult psychotic or depressive symptoms are in line with previous clinical studies in patients and high-risk individuals, suggesting that the characteristics of cognitive development are common within each spectrum. Although some responses using self-report questionnaires are state-dependent, our results could deserve further exploration of the relationship between cognitive profiles and psychological outcomes.

However, several limitations should also be considered. Although demographic characteristics in the NSHD were representative of the population in the survey at age 53 years (Wadsworth *et al.* 2003), those lost to follow-up were more likely to be male, and had lower birth weight and lower cognition scores at ages 8 and 15 years. Lower childhood cognition and adult psychological symptoms are associated with attrition in cohort studies. Similar to the results from clinical studies of schizophrenia and major affective symptoms, people with psychotic experiences had higher all-cause mortality in a prospective study (Sharifi *et al.* 2015). As clinical studies have repeatedly shown these characteristics to be risk factors for schizophrenia, higher attrition for participants with cognitive discrepancy may have led to underestimation of effect size in the relationship between greater verbal developmental lag and psychotic experiences. Attrition of those with low childhood cognitive function may similarly have caused underestimation of effect sizes. However, we have no reason to believe that this would have altered the pattern of associations observed. Second, due to time constraints we did not ask the supplementary questions to each of the five leading PSQ questions, which resulted in some detail about psychotic features being lost. Third, self-report questionnaires may influence responses through misinterpretation or misreading of items, or by transient events. Although the present findings were consistent with clinical case–control studies of disease spectra, repeated measures and interview-based assessment may increase response validity. In addition, the PSQ scale was tested at an item level that may not have allowed sufficient validity, and also was not comparable with the way we used the GHQ-28 as a total score. However, a total score based on four items would not have similar psychometric properties to that based on a 28-item Likert scale. In any case, recent evidence suggests that positive self-reported psychotic experiences are multi-factorial (Therman & Ziermans, 2016). Future research using threshold-level psychotic experiences is needed to confirm the present findings. Fourth, since detailed information for clinical treatment was not available, we were unable to consider whether participants with schizophrenia and major depression in remission were among those who were classed as negative for symptoms. However, this is unlikely to be a significant source of bias, and those with clinical symptoms are only a subgroup of those who self-report symptoms (Jones *et al.* 1994; van Os *et al.* 1997). Although 30 clinical cases of schizophrenia were identified in this cohort, only 11 of these cases completed the GHQ-28 at the 53-year survey (Jones *et al.* 1994). Thus we did not use this classification to test cognitive discrepancy in the psychosis spectrum compared with participants with affective symptoms. Consistent with this, a previous study using this cohort reported that psychotropic medication at age 43 years had little effect on the GHQ-28 score at age 53 years (Colman *et al.* 2008). Fifth, although comparisons with short-form intelligence tests suggest that non-verbal tests at age 8 and 15 years broadly represent non-verbal cognition (Christensen *et al.* 2007), processing speed as measured by, for example, the symbol coding and symbol search subtests was not assessed in this study. We previously showed a deficit of motor development in those who later developed schizophrenia and affective disorders (Jones *et al.* 1994; van Os *et al.* 1997); however, another cohort study did not show a developmental deficit in relation to digit symbol substitution, although a developmental lag through adolescence occurred in people with schizophrenia (Reichenberg *et al.* 2010; Meier *et al.* 2014). In addition, it is a possibility that the participants might use a verbal strategy when performing non-verbal cognitive tests in this study. Further studies are needed of non-verbal cognitive deficit and lag in relation to psychotic and depressive symptoms.

Although cognitive deficits in various domains are evident in patients with schizophrenia (Jones *et al.* 1994; Cannon *et al.* 2002*b*; Fuller *et al.* 2002; Zammit *et al.* 2004; Koenen *et al.* 2009; Welham *et al.* 2009; Reichenberg *et al.* 2010; Khandaker *et al.* 2011; Trotta *et al.* 2015; Bora, 2016) and individuals at high risk of psychosis (Carrion *et al.* 2011; Fusar-Poli *et al.* 2012; Giuliano *et al.* 2012; Lin *et al.* 2013), our results suggest that lower verbal performance is a specific characteristic of the psychosis spectrum. This is in line with another cohort study showing an inverse association between psychotic experiences and intelligence quotient (IQ), especially in verbal IQ after adjusting for non-verbal IQ (Horwood *et al.* 2008). Neuroimaging studies have also shown that individuals with clinical high risk for psychosis had similar brain volume reduction and function in the inferior frontal gyrus, a region responsible for language processing, to patients with first-episode psychosis and chronic schizophrenia (Koike *et al.* 2011; Iwashiro *et al.* 2012; Koike *et al.* 2016).

Of interest, a study using national register data showed that siblings of patients with schizophrenia were more likely to work in creative occupations (e.g. in those with scientific occupations) (Kyaga *et al.* 2011). This association was strengthened after controlling for overall cognition (Kyaga *et al.* 2011). Creativity is more linked to non-verbal cognitive ability than verbal cognition (Cerruti & Wilkey, 2011), suggesting that a discrepancy towards higher non-verbal cognition may be associated with advantage in creative occupations. On the other hand, divergent thinking, related to creativity, is associated with thought disorder in schizophrenia, which is assumed to be derived from verbal cognitive impairment (Crow, 2008; Levy *et al.* 2010).

In contrast, participants with case-level affective symptoms had lower non-verbal cognition at age 8 years and greater relative developmental non-verbal lag, independently of verbal cognition and the other covariates. Consistent with previous studies (Cannon *et al.* 2002*a*; Koenen *et al.* 2009; Reichenberg *et al.* 2010), verbal cognition in those with affective symptoms was not different from that in participants without case-level affective symptoms, even without control for potential confounders, and was relatively high in those with co-morbid psychotic experiences. A meta-analysis suggests that non-verbal executive function, such as visual planning, spatial working memory, visual attention and visual pattern recognition memory, is one of the most prominent cognitive impairments in major depression (Rock *et al.* 2014; Trivedi & Greer, 2014). In addition, these impairments are evident before clinical onset, and show little improvement during clinical course, suggesting a trait factor (Rock *et al.* 2014; Trivedi & Greer, 2014). A recent neuroimaging study showed that a greater verbal–non-verbal discrepancy towards lower non-verbal function was associated with cortical thinning in the rostral part of the prefrontal cortex (Margolis *et al.* 2013), which is one of the regions responsible for executive functions (Badre & D'Esposito, 2009) as well as major depression (Disner *et al.* 2011).

In conclusion, we found that people with adult psychotic experiences and depressive symptoms showed differential verbal–non-verbal cognitive profiles in childhood and adolescence. Our results suggest that these characteristics of cognitive development are common within each disease spectrum. Future prospective studies are needed at different stages across the spectra to elucidate how cognitive profiles are associated with the emergence of psychological symptoms.

## Supplementary material

For supplementary material accompanying this paper visit http://dx.doi.org/10.1017/S0033291717000393. click here to view supplementary material
