# Supplementary material for: Cognitive profiles in childhood and adolescence differ between adult psychotic and affective symptoms: a prospective birth cohort study
Source: Psychol Med. 2017 Oct 9;48(1):11–22. doi: 10.1017/S0033291717000393 (PMC5729848; doi:10.1017/S0033291717000393)
Supplement: Supplementary file 1 [file S0033291717000393sup001.doc]

***Supplementary Table S1.*** *Cognitive characteristics in childhood and adolescence on each affective symptom*

|  | GHQ-28 | |  | |  | |  | |
| --- | --- | --- | --- | --- | --- | --- | --- | --- |
|  | Somatic symptoms | | Anxiety and insomnia | | Social dysfunction | | Severe depression | |
|  | Non-case | Case | Non-case | Case | Non-case | Case | Non-case | Case |
|  | n=2,195  (92.4%) | n=180  (7.6%) | n=2,165  (91.0%) | n=214  (9.0%) | n=2,158  (90.8%) | n=219  (9.2%) | n=2,194  (92.3%) | n=182  (7.7%) |
| Male, n (%) | **1096**  **(94.6%)** | **62*****  **(5.4%)** | **1084**  **(93.6%)** | **74*****  **(6.4%)** | **1081**  **(93.5%)** | **75*****  **(6.5%)** | **1086**  **(94.0%)** | **69*****  **(6.0%)** |
| Cognition at age 8 |  |  |  |  |  |  |  |  |
| Verbal Domain | 0.06  (0.97) | -0.08  (1.02) | 0.06  (0.98) | -0.02  (0.96) | 0.06  (0.98) | -0.04  (0.93) | **0.07**  **(0.98)** | **-0.16****  **(0.96)** |
| Non-verbal domain | **0.09**  **(0.95)** | **-0.06***  **(0.97)** | **0.09**  **(0.94)** | **-0.07***  **(1.06)** | 0.08  (0.94) | -0.00  (1.03) | **0.10**  **(0.93)** | **-0.18*****  **(1.15)** |
| Cognition at age 15 |  |  |  |  |  |  |  |  |
| Verbal Domain | **0.10**  **(0.95)** | **-0.05***  **(0.95)** | **0.10**  **(0.94)** | **-0.05***  **(0.98)** | **0.10**  **(0.95)** | **-0.04***  **(0.96)** | **0.11**  **(0.94)** | **-0.14*****  **(1.01)** |
| Non-verbal domain | **0.08**  **(0.94)** | **-0.20*****  **(0.92)** | **0.08**  **(0.93)** | **-0.18*****  **(1.05)** | **0.08**  **(0.94)** | **-0.14*****  **(0.99)** | **0.09**  **(0.93)** | **-0.21*****  **(1.02)** |
| Developmental lag from age 8 to 15^1^ |  |  |  |  |  |  |  |  |
| Verbal Domain | 0.038  (0.699) | 0.021  (0.695) | 0.044  (0.702) | -0.031  (0.662) | 0.040  (0.702) | 0.008  (0.668) | 0.038  (0.701) | 0.027  (0.680) |
| Non-verbal Domain | -0.002  (1.030) | -0.139  (1.030) | -0.003  (1.030) | -0.106  (1.079) | 0.001  (1.040) | -0.137  (0.986) | -0.009  (1.030) | -0.035  (1.133) |

Mean (SD). Bold showed significant difference by chi square test for sex, and t test for the other variables (* p < .05, ** p < .01, *** p < .001).

Abbreviations: GHQ-28, the 28 item version of the General Health Questionnaire.

1) The differences of Z scores from age 8 to 15 for verbal and non-verbal domains (score at age 15 years – 8 years). A positive score represents increase of developmental lag from mean.

**Supplementary Table S2.** *Details in association of cognitive characteristics in childhood and adolescence with each psychotic experience and affective symptoms*

|  | PSQ |  |  |  |  |  |  |  |  |  |  |  |
| --- | --- | --- | --- | --- | --- | --- | --- | --- | --- | --- | --- | --- |
|  | Thought Interference | | | Persecution | | | Strange Experience | | | Hallucination | | |
|  | Yes (n=433) vs. No or unsure (n=1,945) | | | Yes (n=508) vs. No or unsure (n=1,870) | | | Yes (n=296) vs. No or unsure (n=2,083) | | | Yes (n=88) vs. No or unsure (n=2,289) | | |
| Independent variables | Unadjusted | Adjusted 1 | Adjusted 2 | Unadjusted | Adjusted 1 | Adjusted 2 | Unadjusted | Adjusted 1 | Adjusted 2 | Unadjusted | Adjusted 1 | Adjusted 2 |
| Cognition at age 8 |  |  |  |  |  |  |  |  |  |  |  |  |
| Verbal Domain | **0.78*****  **[0.70 0.87]** | **0.76*****  **[0.67 0.86]** | **0.81***  **[0.68 0.97]** | 0.94  [0.85 1.04] | 0.93  [0.83 1.04] | 0.92  [0.78 1.09] | **0.84****  **[0.74 0.96]** | 0.88  [0.76 1.01] | **0.79***  **[0.64 0.97]** | **0.68*****  **[0.54 0.85]** | **0.67****  **[0.51 0.87]** | **0.55****  **[0.38 0.80]** |
| Non-verbal Domain | **0.83*****  **[0.75 0.92]** | **0.86****  **[0.77 0.96]** | 0.91  [0.76 1.10] | 0.94  [0.85 1.04] | 0.96  [0.86 1.08] | 1.00  [0.84 1.19] | **0.84****  **[0.74 0.95]** | **0.88***  **[0.77 0.999]** | 0.99  [0.80 1.22] | **0.72****  **[0.59 0.89]** | **0.70****  **[0.56 0.88]** | 1.02  [0.70 1.47] |
| Developmental lag  from age 8 to 15 |  |  |  |  |  |  |  |  |  |  |  |  |
| Verbal Domain | 1.15  [0.99 1.34] | **1.19***  **[1.02 1.39]** | 1.07  [0.88 1.31] | 1.04  [0.90 1.19] | 1.04  [0.90 1.20] | 0.99  [0.83 1.20] | **0.82***  **[0.69 0.98]** | **0.83***  **[0.69 0.995]** | **0.71****  **[0.56 0.90]** | 0.88  [0.65 1.19] | 0.79  [0.58 1.09] | **0.52****  **[0.34 0.79]** |
| Non-verbal Domain | 1.03  [0.93 1.14] | 1.02  [0.92 1.13] | 0.93  [0.80 1.08] | 1.02  [0.92 1.12] | 1.01  [0.91 1.11] | 1.00  [0.87 1.14] | 1.02  [0.91 1.15] | 1.02  [0.90 1.15] | 1.04  [0.88 1.24] | 1.11  [0.91 1.36] | 1.16  [0.94 1.44] | 1.23  [0.91 1.67] |

(Continued)

|  | PSQ |  |  | GHQ-28 |  |  |  |  |  |  |  |  |
| --- | --- | --- | --- | --- | --- | --- | --- | --- | --- | --- | --- | --- |
|  | Any of psychotic experiences | | | GHQ-28 caseness | | | Somatic symptoms | | | Anxiety and insomnia | | |
|  | Case (n=772) vs. non-case (n=1,607) | | | Case (n=453) vs. non-case (n=1,913) | | | Case (n=180) vs. non-case (n=2,195) | | | Case (n=214) vs. non-case (n=2,165) | | |
| Independent variables | Unadjusted | Adjusted 1 | Adjusted 2 | Unadjusted | Adjusted 1 | Adjusted 2 | Unadjusted | Adjusted 1 | Adjusted 2 | Unadjusted | Adjusted 1 | Adjusted 2 |
| Cognition at age 8 |  |  |  |  |  |  |  |  |  |  |  |  |
| Verbal Domain | **0.83*****  **[0.76 0.91]** | **0.82*****  **[0.74 0.91]** | **0.82****  **[0.70 0.94]** | 0.99  [0.90 1.10] | 0.93  [0.83 1.06] | 1.05  [0.88 1.26] | 0.86  [0.74 1.01] | **0.82***  **[0.68 0.99]** | 0.96  [0.74 1.25] | 0.92  [0.79 1.06] | 0.88  [0.75 1.04] | 0.98  [0.77 1.25] |
| Non-verbal Domain | **0.89****  **[0.81 0.97]** | 0.91  [0.83 1.00] | 0.99  [0.85 1.15] | 0.95  [0.86 1.06] | 0.95  [0.84 1.06] | **0.77****  **[0.64 0.93]** | **0.85***  **[0.73 0.996]** | 0.85  [0.72 1.00] | **0.72***  **[0.55 0.95]** | **0.85***  **[0.73 0.98]** | **0.84***  **[0.84 0.98]** | **0.73***  **[0.57 0.93]** |
| Developmental lag  from age 8 to 15 |  |  |  |  |  |  |  |  |  |  |  |  |
| Verbal Domain | 1.09  [0.96 1.23] | 1.09  [0.96 1.24] | 0.98  [0.83 1.15] | 0.93  [0.80 1.08] | 0.98  [0.84 1.14] | 1.11  [0.91 1.36] | 0.97  [0.78 1.20] | 1.03  [0.82 1.30] | 1.11  [0.82 1.49] | 0.86  [0.70 1.05] | 0.90  [0.73 1.11] | 0.95  [0.72 1.24] |
| Non-verbal Domain | 1.02  [0.94 1.11] | 1.01  [0.92 1.10] | 0.98  [0.87 1.10] | **0.86****  **[0.78 0.95]** | **0.86****  **[0.78 0.96]** | **0.74*****  **[0.64 0.86]** | 0.88  [0.76 1.02] | 0.91  [0.77 1.06] | **0.74****  **[0.59 0.93]** | 0.91  [0.79 1.04] | 0.93  [0.81 1.08] | **0.80***  **[0.65 0.97]** |

(Continued)

|  | GHQ-28 |  |  |  |  |  |
| --- | --- | --- | --- | --- | --- | --- |
|  | Social dysfunction | | | Severe depression | | |
|  | Case (n=219) vs. non-case (n=2,158) | | | Case (n=182) vs. non-case (n=2,194) | | |
| Independent variables | Unadjusted | Adjusted 1 | Adjusted 2 | Unadjusted | Adjusted 1 | Adjusted 2 |
| Cognition at age 8 |  |  |  |  |  |  |
| Verbal Domain | 0.89  [0.77 1.03] | 0.88  [0.75 1.04] | 0.94  [0.74 1.19] | **0.78****  **[0.66 0.91]** | **0.81***  **[0.67 0.97]** | 0.99  [0.76 1.29] |
| Non-verbal Domain | 0.91  [0.79 1.06] | 0.94  [0.81 1.10] | 0.86  [0.67 1.10] | **0.75*****  **[0.65 0.87]** | **0.79****  **[0.67 0.92]** | **0.70****  **[0.53 0.92]** |
| Developmental lag  from age 8 to 15 |  |  |  |  |  |  |
| Verbal Domain | 0.94  [0.77 1.14] | 1.03  [0.84 1.27] | 1.07  [0.81 1.40] | 0.98  [0.79 1.21] | 1.08  [0.86 1.35] | 1.12  [0.83 1.51] |
| Non-verbal Domain | 0.88  [0.77 1.01] | 0.91  [0.79 1.04] | **0.82***  **[0.67 0.999]** | 0.98  [0.84 1.13] | 1.02  [0.88 1.19] | 0.82  [0.66 1.02] |

OR [95%CI]. Bold showed significant coefficient (* p < .05, ** p < .01, *** p < .001). Adjusted model 1 was controlled for confounding variables (sex, birth weight, birth order, mother’s education, and social class of origin). Adjusted model 2, which was summarized in table 2, was controlled for confounding variables and the other 3 independent variables.

**Supplementary Table S3.** *Details in cognitive characteristics in childhood and adolescence by existence of psychotic experience and/or affective symptoms*

|  | PE only (n=194) vs. no symptom (n=1,714) | | | AFF only (n=315) vs. no symptom (n=1,714) | | | Both (n=138) vs. no symptom (n=1,714) | | | AFF only (n=315) vs. PE only (n=194) | | |
| --- | --- | --- | --- | --- | --- | --- | --- | --- | --- | --- | --- | --- |
| Independent variables | Unadjusted | Adjusted 1 | Adjusted 2 | Unadjusted | Adjusted 1 | Adjusted 2 | Unadjusted | Adjusted 1 | Adjusted 2 | Unadjusted | Adjusted 1 | Adjusted 2 |
| Cognition at age 8 |  |  |  |  |  |  |  |  |  |  |  |  |
| Verbal Domain | 0.86  [0.74 1.00] | 0.91  [0.76 1.09] | 0.79  [0.61 1.02] | 1.07  [0.95 1.21] | 1.01  [0.87 1.16] | 1.21  [0.98 1.49] | **0.79***  **[0.66 0.95]** | **0.76****  **[0.62 0.93]** | **0.72***  **[0.53 0.97]** | **0.78***  **[0.65 0.95]** | 0.87  [0.69 1.10] | **0.64***  **[0.45 0.91]** |
| Non-verbal Domain | 0.90  [0.77 1.05] | 0.95  [0.80 1.12] | 1.12  [0.86 1.46] | 1.04  [0.92 1.19] | 1.03  [0.89 1.18] | **0.73****  **[0.58 0.91]** | **0.77****  **[0.65 0.91]** | **0.78****  **[0.65 0.94]** | 0.85  [0.63 1.15] | 0.86  [0.71 1.04] | 0.92  [0.73 1.15] | 1.40  [0.98 2.01] |
| Developmental lag  from age 8 to 15 |  |  |  |  |  |  |  |  |  |  |  |  |
| Verbal Domain | 0.94  [0.76 1.15] | 0.89  [0.72 1.11] | 0.75  [0.57 1.00] | 0.99  [0.83 1.17] | 1.03  [0.86 1.23] | **1.33***  **[1.05 1.68]** | 0.79  [0.62 1.01] | 0.84  [0.65 1.09] | **0.70***  **[0.50 0.99]** | 0.94  [0.72 1.23] | 0.86  [0.64 1.16] | **0.59****  **[0.40 0.86]** |
| Non-verbal Domain | 1.05  [0.91 1.21] | 1.03  [0.89 1.20] | 1.12  [0.91 1.38] | **0.81*****  **[0.72 0.92]** | **0.80*****  **[0.71 0.91]** | **0.66*****  **[0.55 0.79]** | 0.97  [0.82 1.15] | 0.99  [0.83 1.18] | 0.93  [0.72 1.18] | **1.29****  **[1.08 1.53]** | 1.17  [0.96 1.44] | **1.50****  **[1.13 2.01]** |

OR [95%CI]. Bold showed significant coefficient (* p < .05, ** p < .01, *** p < .001). Participants were grouped into psychotic experience (PE) only (defined by existence of one or more items of strange experience and hallucination), affective symptoms (AFF) only, both, and no symptom groups. Adjusted model 1 was controlled for confounding variables (sex, birth weight, birth order, mother’s education, and social class of origin). Adjusted model 2, which was summarized in table 3, was controlled for confounding variables and the other 3 independent variables.
